# Caring for Children with an Autism Spectrum Disorder: Factors Associating with Health- and Care-Related Quality of Life of the Caregivers

**DOI:** 10.1007/s10803-021-05336-7

**Published:** 2021-11-01

**Authors:** Leontine W. ten Hoopen, Pieter F. A. de Nijs, Jorieke Duvekot, Kirstin Greaves-Lord, Manon H. J. Hillegers, Werner B. F. Brouwer, Leona Hakkaart-van Roijen

**Affiliations:** 1 Department of Child and Adolescent Psychiatry/Psychology, Erasmus MC, Sophia Children’s Hospital, Rotterdam, P.O. Box 2040, 3000 CA Rotterdam, The Netherlands; 2 Erasmus School of Health Policy & Management, Erasmus University Rotterdam, Burgemeester Oudlaan 50, 3062 PA Rotterdam, The Netherlands; 3 Yulius Mental Health, P.O. Box 753, 3300 AT Dordrecht, The Netherlands; 4 Jonx Autism Team Northern-Netherlands, Lentis Mental Health, Laan Corpus Den Hoorn 102-2, 9728 JR Groningen, The Netherlands

**Keywords:** Autism spectrum disorder (ASD), Children, Caregiver, Health-related quality of life, CarerQol, EQ-5D

## Abstract

This study investigated the association of child, caregiver, and caregiving measurements with the quality of life (QoL) in 81 caregivers (mostly parents) of clinically referred children with autism spectrum disorder (ASD). We used the EuroQol five-dimensional (EQ-5D) questionnaire and the care-related QoL questionnaire (CarerQol) to respectively assess health-related QoL and care-related QoL. Health-related QoL was associated with the caregiver’s internalizing problems and adaptive coping, explaining 38% of the variance. Parenting stress and adaptive coping were associated with the care-related QoL and explained 60% of the variance. Child variables were not associated with the caregiver’s health- and care-related QoL if caregiver and caregiving variables were taken into account. Findings indicate the importance of the caregiver’s mental health, coping, and parenting stress in caring for children with ASD.

**Supplementary Information:**

The online version contains supplementary material available at 10.1007/s10803-021-05336-7.

Caregivers—often the parents—of children with autism spectrum disorder (ASD) report a lower health-related quality of life (QoL), compared to general population norms (Khanna et al., 2011; Kuhlthau et al., 2014) or to caregivers of typically developing, chronically ill, or disabled children (Kheir et al., 2012; Mugno et al., 2007). These caregivers have a higher risk of mental health problems, such as stress, depression, and anxiety (Allik et al., 2006; Falk et al., 2014). Fairthorne et al. (2014) even found a higher chance of dying at a young age in mothers of children with ASD. Qualitative data support the impression that parenting a child with ASD is demanding and affects the caregiver’s health-related QoL negatively (Kuhlthau et al., 2014). Besides these health problems, caregivers may also experience many challenges because of the caring, such as problems combining the care with other daily activities or relational problems with the child they care for (Hoefman et al., 2014).

While most studies have focused on the negative aspects of caring for a child with ASD, caregivers may experience positive aspects simultaneously. Kayfitz et al. (2010) reported parents’ positive experiences in raising their children with autism and discussed the possible positive effect on resilience. In addition, Fong et al. (2021) found more satisfaction with informal support to contribute positively to family resilience. Hoefman et al. (2014) also found positive aspects of caring, such as fulfillment and experienced support in caring, to impact the care-related QoL of caregivers. This information indicates that caring can affect the caregiver’s QoL, both positively and negatively. To study which factors influence the caregiver’s QoL, and potentially enhance caregiver outcomes, we have to include both positive and negative QoL aspects.

In previous studies in children with ASD, the caregiver’s QoL was found to be associated with several child, caregiver, and caregiving situation variables (Vasilopoulou & Nisbet, 2016). Child variables, such as age (Tung et al., 2014), behavior problems (Khanna et al., 2011; McStay et al., 2014), emotional problems (Bourke-Taylor et al., 2012; Totsika et al., 2011), and autism severity (Khanna et al., 2011; Tung et al., 2014) seemed to be negatively associated with the caregiver’s QoL. However, this latter finding was not confirmed in other studies (Lee et al., 2009; McStay et al., 2014). Concerning caregiver’s variables, female sex (Allik et al., 2006; McStay et al., 2014) and stress (Lee et al., 2009; Tung et al., 2014) were negatively related to the caregiver’s QoL, whereas self-efficacy (Bourke-Taylor et al., 2012), perceived satisfaction with the marital relationship (Hartley et al., 2011), and social support (Khanna et al., 2011; McStay et al., 2014) seemed to be positively related. Inconclusive results were found for the association between caregiver’s coping and QoL (Khanna et al., 2011; Lee et al., 2009). Especially coping focused on problem-solving seemed more effective than coping focused on emotions in improving the well-being of mothers (Smith et al., 2008). Caregivers’ QoL was also positively associated with caregiving situation variables, such as families with more children (Lee et al., 2009), and paid employment of the caregiver (Bourke-Taylor et al., 2012). So, while information about variables associated with the caregiver’s QoL is essential for understanding and potentially improving it, the evidence on these variables is currently limited.

Nonetheless, the impact of caring on the caregiver’s QoL is crucial because of the continuous reciprocal interaction process between children and their caregivers. Caring for a child with ASD may impact the caregiver’s QoL, with effects on the caregiver’s interaction with the child, which in turn may influence the child’s well-being. Rodriguez et al. (2019) already showed such transactional effects between children with ASD and their caregivers, for example, showing associations between parenting stress and behavior problems as well as ASD symptoms of the children. Next to these transactional effects in families, studies showed that caregiver’s involvement and well-being are essential in successfully applying interventions and treatment aimed at children with ASD (Osborne et al., 2008; Volkmar et al., 2014).

Because in most studies the caregiver’s QoL was measured with a health-related QoL measure, the findings and impact of the caring focus on the subjective self-evaluation of the caregiver’s health aspects. In a few studies, the care-related QoL in caregivers raising children with ASD was included (Hoefman et al., 2014; Ten Hoopen et al., 2020). The findings from these studies suggest each concept of QoL to provide unique information about the impact of caring for a child with ASD. Therefore, to avoid a one-sided look, we believe it is important to include both perspectives on caregiver’s QoL, studying the relevant variables in improving caregiver outcome.

This study aimed to expand on previous literature by exploring associations of the caregiver’s QoL with an extensive but structured set of child, caregiver, and caregiving situation variables in a well-defined group of clinically referred children with an ASD classification. Based on previous study results, general characteristics (i.e., sex and age) and problem scores (i.e., social impairments, emotional and behavior problems) of the children and their caregivers and some caregiving situation variables were included. We were especially interested in the potential protective factors of caregivers’ QoL and included aspects like coping, personal growth, partner-relationship and social support, and caregivers' employment. Both health-related QoL and care-related QoL were investigated. Because of these different perspectives on the caregiver’s QoL, we expected both to be associated with other independent variables. We hypothesized that the caregiver’s health-related QoL was associated with the caregiver’s general and problem variables because of the aspects of health-related QoL. We expected the care-related QoL to be associated with child and caregiving variables because of the caring elements involved. The results may provide information relevant to understanding and, ultimately, improving the QoL of caregivers of children with ASD.

## Methods

### Data Collection

The data collection took place as part of the “Social Spectrum Study”, which is a prospective multicenter study focused on individual, familial, and societal characteristics of clinically referred children with autistic traits (Duvekot et al., 2017). Before collecting the data, the Medical Ethics Committee of the Erasmus Medical Center and the participating centers for mental health care approved this study (MEC-2011-078). For all assessments, we obtained written informed consent from the parents/caregivers of the participating children.

**Fig. 1:**
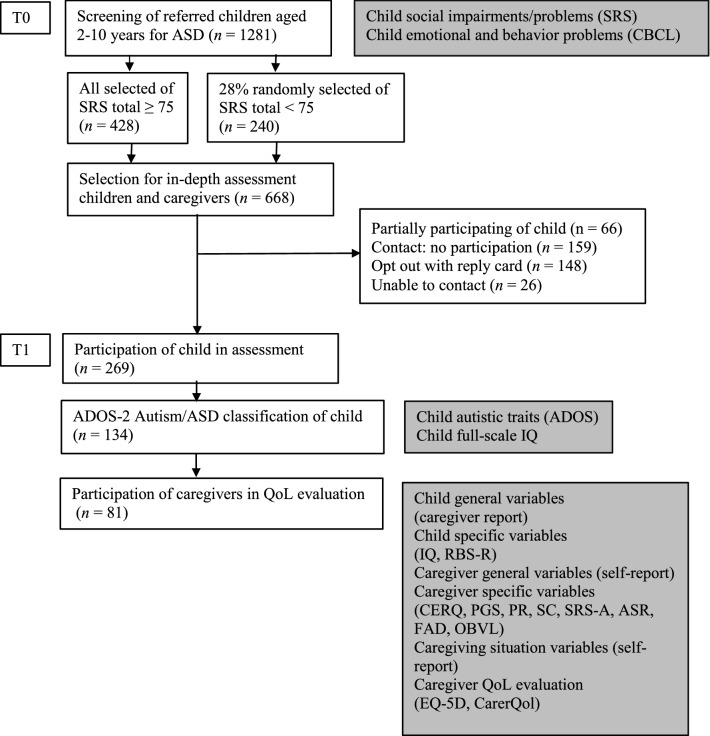
Flowchart of the study design with instruments at each time point. *ASD* autism spectrum Disorder, *SRS* social responsiveness scale, *CBCL* child behavior checklist, *ADOS* autism diagnostic observation schedule second edition, *IQ* intelligence quotient, *RBS-R* repetitive behavior scale-revised, *CERQ* cognitive emotion regulation questionnaire, *PGS* personal growth scale, *PR* partner relationship, *SC* social contacts, *SRS-A* social responsiveness scale-adults, *ASR* adult self-report, *FAD* family assessment device, *OBVL* opvoedingsbelasting vragenlijst [Parenting Stress Questionnaire], *EQ-5D* EuroQol five-dimensional quality of life questionnaire, *CarerQol* care-related quality of life questionnaire

For the study, we selected children (aged 2–10 years) with a high likelihood of ASD out of all referrals to six child and adolescent mental health (CAMH) centers in the South-West of the Netherlands. Because the children were referred for all kinds of developmental, behavioral, and emotional problems, we used the parent-reports on the Social Responsiveness Scale (SRS; Constantino & Gruber, 2012) for the selection of children with a high likelihood of ASD. This selection phase took a half year at each site, in the period from April 2011 till July 2012. With an oversampling design (Duvekot et al., 2017), we found 668 children with a high likelihood of having ASD. Using the Autism Diagnostic Observation Schedule, second edition (ADOS-2, Lord et al., 2012), we identified 134 children with an ADOS-2 classification of ‘Autism’ or ‘Autism Spectrum Disorder’ (further referred to as *ASD* or an *ASD classification*). Figure 1 shows that for the present study, 81 (60%) caregivers of these 134 children completed health- and care-related self-reports, as well as questionnaires on child and caregiver general characteristics, child and caregiver emotional and behavior problems, child and caregiver psychopathology, and caregiving situation characteristics. The participating caregiver was the informal caregiver most involved in raising the child (i.e., the primary caregiver), predominantly a parent. All primary caregivers reported their relationship with the child (e.g., biological parent, foster parent, adoption parent, grandparent, step-parent, or otherwise). Formal caregivers, such as psychologists, therapists, social workers, or physicians, were not included as respondents in this study.

### Child Measurements

#### Child General Characteristics

General information on the child included sex, age, ethnic background, and the position of the child in the family. If no valid IQ assessment was performed during the past two years, age-appropriate IQ assessment was carried out by a trained psychologist (Duvekot et al., 2017).

#### Child Problems and Psychopathology

Internalizing and externalizing problems of the child were reported by the caregiver on the Child Behavior Checklist (CBCL; Achenbach & Rescorla, 2000, 2001), with higher scores meaning more child problems. Because of different age-dependent versions of the CBCL for children aged 1.5–5 years (99 items, *n* = 34; 45%) versus children aged 6–18 years (118 items, *n* = 42; 55%), we calculated the *T* scores of total internalizing problems and total externalizing problems to ensure comparability of the scores. Pandolfi et al., (2009, 2014) confirmed fairly good psychometric properties of the CBCL in ASD samples (CBCL 1,5–5: α ranging from 0.49 to 0.83; CBCL 6–18: > 0.70). In our study, we found an acceptable internal consistency (CBCL 1,5–5: α = 0.77; CBCL 6–18: α = 0.81).

Social problems or impairments of the child were reported by the caregiver on the 65 items of the Social Responsiveness Scale (SRS; Constantino & Gruber, 2012; Roeyers et al., 2011), with higher scores meaning more child problems. We converted total raw scores of the broadly similar SRS versions for preschool children (2.5 to 4 years; *n* = 12; 15%) and school-age children (aged ≥ 4 years; *n* = 69; 85%) into *T*-scores, based on norms for gender, age, and rater type (Constantino & Gruber, 2012). Roeyers et al. (2011) found a high internal consistency in the Dutch versions of the SRS (*α* ranging from 0.92 to 0.95), similar to the high internal consistency in our study (α = 0.92).

Repetitive behaviors/interests of the child were assessed by the caregiver on the 43 items of the Repetitive Behavior Scale-Revised (RBS-R; Bodfish et al., 1999), with a higher total sum score meaning more problems. Mirenda et al. (2010) confirmed the utility of the RBS-R in young children with ASD. We found a high internal consistency in our study (α = 0.94).

Autistic traits were assessed with the Autism Diagnostic Observation Schedule-2 (ADOS-2; Lord et al., 2012) by a trained and certified professional. Next to an ASD classification, we calculated the standardized calibrated severity scores (CSS, range 1–10), indicating autism severity for the child’s age and expressive language level (Gotham et al., 2009). A higher CSS meant more autism severity. Good psychometric properties were shown before (Lord et al., 2012), with a good interrater agreement (on items ranging from 88 to 92%, and on classification ranging from 92 to 98%).

### Caregiver Measurements

#### Caregiver General Characteristics

The collected general information from the caregiver included sex, age, and highest attained educational level (self-report).

The caregiver’s personal growth was measured by a five-level answer on the five-item Personal Growth Scale (PGS; Kraaij et al., 2008). Higher scores indicated more perceived personal positive changes in the appreciation of personal life and strength with stressful events. In our study, this scale was found to have a high internal consistency (α = 0.93).

The satisfaction with—and perceived support from—the partner-relationship (PR) and social contacts (SC) were each reported using four-level answers on the five-item subscales of the Family Functioning Questionnaire reported by Parents (VGFO; *Vragenlijst Gezinsfunctioneren volgens Ouders*; Veerman et al., 2012). Higher scores reflected more satisfaction or more perceived support. Each of these two subscales turned out te be internally consistent in our study (PR: α = 0.91; SC: α = 0.78).

The caregiver’s coping was assessed with subscales of the Cognitive Emotion Regulation Questionnaire (CERQ; Garnefski & Kraaij, 2007), using the four-item scale ‘putting into perspective’ as an index of *adaptive* coping and the four-item scale ‘ruminating’ as an index of *maladaptive* coping. Higher scores on the five-level answers meant more adaptive or more maladaptive coping. In our study, these CERQ subscales were found to have a good internal consistency (CERQ adaptive coping: α = 0.85; maladaptive coping α = 0.81).

#### Caregiver Problems and Psychopathology

Caregivers themselves provided information about their problems and psychopathology. Social problems or impairments were measured with the total score on 64 items of the Adult version of the Social Responsiveness Scale”, which was converted into a *T*-score, based on self-report population norms (SRS-A; Constantino & Gruber, 2012); higher scores were indicating more problems. The SRS-A has been reported internally consistent (*α* ranging from 0.71 in adults without ASD to 0.89 in adults with ASD, Bölte, 2012). In our study, a good internal consistency was found (α = 0.93).

Internalizing and externalizing problems were scored by the caregivers themselves on the Adult Self-Report three-level scale (ASR; Achenbach & Rescorla, 2003), with higher scores on the 120 items indicating more problems. Internal consistency was reported to range between 0.51 and 0.97 (Achenbach & Rescorla, 2003). In our study, the internal consistency of the ASR also proved to be good (α = 0.84).

Parenting stress was reported by the caregivers with four-level answers on the parenting stress questionnaire (OBVL; *Opvoedingsbelastingvragenlijst*; Vermulst et al., 2012), with a higher total score reflecting more experienced stress. The 34 items of the OBVL cover five subscales (‘Parent–child relation problems’, ‘Parenting problems’, ‘Depressive mood’, ‘Role limitation’, ‘Health problems’), and add up to a total sum score. In our study, the instrument proved to have good internal consistency (α = 0.94).

### Caregiving Situation Measurements

General information on the caregiving situation included the total number of children in the family, one- or two-parent household, caregiver’s employment, and the number of weekly working hours of employment. If the caregiver indicated to have a partner, also the partner’s employment and their number of weekly working hours were reported by the caregiver (proxy report). In this study, no income data were included.

Family functioning was scored by the caregivers with four-level answers on the General Functioning Scale of the Family Assessment Device (FAD; Epstein et al., 1983). Higher scores on the twelve items indicated more family problems. This scale was found to have a good internal consistency in our study (α = 0.87).

### Health-Related Quality of Life (EQ-5D)

Caregivers reported the health-related QoL on the EuroQoL Five Domain Health Questionnaire (EQ-5D, Brooks, 1996; The EuroQoL Group, 1990; www.euroqol.org) with three-level-answers (no problems, some or moderate, and extreme problems) on the five domains: mobility, self-care, usual activities, pain/discomfort, and anxiety/depression. To calculate the EQ-5D utility score of the caregiver, we combined this score with a pre-existing set of the Dutch general population preference values to be in this health state (Lamers et al., 2006). With this algorithm, the EQ-5D utility scores range from 0 (state equal to being dead) to 1 (state of perfect health). However, a negative utility score is possible for health states which are considered to be worse than dead (e.g., extreme mental or physical pain). The advantage of this method is the comparability of health-related QoL measurements across conditions and samples. As a standard part of the EQ-5D, caregivers also assessed their health on the visual analog scale (EQ-VAS), ranging from 0 (worst imaginable health state) to 100 (best imaginable health state). We found an internal consistency of 0.64 (Cronbach’s α), which is less than the threshold of 0.70. All corrected item-total correlations were higher than the minimum norm of 0.20. This is consistent with previous findings by Khanna et al., 2013).

### Care-Related Quality of Life (CarerQol)

Caregivers reported the impact of caring on the Care-Related Quality of Life Instrument (CarerQol, Brouwer et al., 2006) with three-level answers (no, some, and a lot) on seven dimensions. This instrument consists of two positive care-related dimensions: ‘*fulfillment* with carrying out the care tasks’ and ‘*support* with informal care tasks from family, friends, neighbors, and acquaintances when needed’ and five negative care-related dimensions: ‘*relational problems* with the care recipient’, ‘*mental health problems*’, ‘*problems combining the care* with daily activities’, ‘*financial problems* because of the care tasks’, and ‘*physical health problems*’. The combination of the answer levels on the seven dimensions represents a set of so-called caring situations, being valued by individuals to be in that specific caring situation. We combined the scored caring situation with a pre-existing set of Dutch general population preference values to be in this caring situation (Hoefman et al., 2011) to calculate the so-called CarerQol tariffs, ranging from score 0 (worst caring situation) to 100 (best caring situation). Using this method, comparing of care-related QoL across conditions and samples is possible. The caregivers also rated their happiness on the visual analog scale (VAS; Brouwer et al., 2006) as a part of the CarerQol, ranging from completely unhappy (0) to completely happy (10). Clinical validation studies showed the suitability of the CarerQol in measuring the impact on caregivers of caring for children with developmental problems, such as ASD (Hoefman et al., 2014; Payakachat et al., 2011). In our study, the reliability in terms of internal consistency was acceptable with a Cronbach’s *α* of 0.71.

### Statistical Analyses

To answer the research questions of this study, we analyzed data of the caregivers of children with an ASD classification, a completed EQ-5D, *and* a completed CarerQol. Possible differences between the groups of 81 children *with* and 53 children (134 minus 81, see Fig. 1) *without* the caregiver’s QoL reports were tested with *t*-tests. To provide insight into the study sample characteristics, we calculated means with standard deviations (continuous variables) or frequencies with percentages (categorical variables). With proportions of missing values ranging between 0 and 8%, we tested if values were missing completely at random (Little’s MCAR test). In the analyses, we treated missing values with listwise deletion.

Statistical analyses were conducted in two phases. In the first phase, we explored the relations between the caregiver’s health-related QoL (EQ-5D utility score) and care-related QoL (CarerQol tariff) on the one hand, and all collected general child and caregiver variables, child and caregiver psychopathology variables, and caregiving situation variables on the other hand, with univariate single-variable regression models (Tables S1–S2). In the second phase, we further explored which variables were significantly associated with the health-related and care-related QoL of the caregivers. Given the exploratory nature of the study, we included all significant child, caregiver, and caregiving situation variables (significance level of 0.05) of the single-variable regression models in two multivariable stepwise regression models, one with the caregiver’s health-related QoL as the dependent variable, and one with the caregiver’s care-related QoL as the dependent variable. To minimize the risk of possible multicollinearity between variables and confounding, we tested and met the underlying assumptions of the regression models, and we used a stepwise procedure in the analyses. To test the robustness of the results, we also performed the ‘forced entry’ multivariable regression analysis in the second phase. We used an alpha of 0.01 as a value for significance in these analyses, in order to balance the risk of type I and type II errors with the sample size. SPSS version 24.0 was used to perform the analyses.

## Results

### Sample Characteristics

Table 1 shows the child, caregiver, and caregiving situation characteristics of the study sample. The caregivers were mostly the child’s biological parent (98%), female (90%), and employed (75%). On average, they were 37.2 years (*SD* 5.2) old. About 73% had a medium or higher educational level. The mean caregivers’ EQ-5D utility score was 0.84 (*SD* 0.17), and the mean CarerQol tariff was 77.33 (*SD* 16.44). Most of the children were male (83%), of Dutch ethnicity (79%), and living in a two-parent household (93%). On average, they were 6.1 years old (*SD* 2.26). The children had a mean full-scale IQ of 94.86 (*SD* 18.75), and a mean *T-*score on the parent-reported SRS of 77.31(*SD* 11.67). Children with caregiver’s reports of QoL were more likely to be of Dutch ethnicity (*p* = 0.036), and to be living with both caregivers (*p* < 0.0001), than children without these reports. Missing values were present in less than half of all variables, and not exceeding 8% (Table 1). Employment data of the caregiver’s partners had the most missing values, mainly because of absent partners. Little’s test showed that the missing values were distributed completely at random (*p* = 0.68).

**Table 1:** Child, caregiver, and caregiving situation characteristics of the study sample

	Characteristics	Instruments	Study sample (*n* = 81) *M* (*SD*)/*n* (%)	Missing values *n* (%)
Child general characteristics				
	Sex (% boys)	Proxy report	67 (82.7)	–
	Age (years)	Proxy report	6.06 (2.26)	–
	Ethnicity (% Dutch)	Proxy report	64 (79.0)	–
	Ranking in the family (% first child)	Proxy report	44 (55.0)	1 (1.2)
	Full-scale IQ	Wechsler tests	94.86 (18.75)	7 (8.6)
Child problem and psychopathology characteristics				
	Social problems (*T*-score)	SRS	77.31 (11.67)	–
	Autistic traits (ADOS-2 CSS)	ADOS-2	6.36 (1.81)	–
	Autism(% classification)	ADOS-2	52 (64.2)	–
	Internalizing problems (*T*-score subscale)	CBCL	67.11 (8.94)	5 (6.2)
	Externalizing problems (*T*-score subscale)	CBCL	66.26 (10.92)	5 (6.2)
	Repetitive behavior (total score)	RBS-R	20.20 (14.66)	–
	Disability (% reported)	Proxy report	18 (22.2)	–
Caregiver general characteristics				
	Sex (% female)	Self-report	73 (90.1)	–
	Age (years)	Self-report	37.2 (5.2)	–
	Biological parent of the child (%)	Self-report	78 (97.5)	–
	Education level (%)	Self-report	–	1 (1.2)
	Low	Self-report	22 (27.2)	–
	Medium	Self-report	38 (46.9)	–
	High	Self-report	21 (25.9)	–
	Adaptive coping (total score subscale)	CERQ	12.51 (3.99)	–
	Maladaptive coping (total score subscale)	CERQ	8.64 (3.30)	–
	Personal growth (total score)	PGS	19.43 (4.81)	–
	Partner-relationship support (total score)	PR	16.08 (3.56)	7 (8.6)
	Social support (total score)	SC	15.54 (3.06)	–
	Parenting stress (total score)	OBVL	61.07 (14.67)	–
	Health-related QoL (utility score)	EQ-5D	0.84 (0.17)	–
	Care-related QoL (tariff)	CarerQol	77.33 (16.44)	5 (6.2)
Caregiver problem and psychopathology characteristics				
	Social problems (*T*-score)	SRS-A	48.78 (8.57)	–
	Internalizing problems (*T*-score subscale)	ASR	52.21 (13.04)	1 (1.2)
	Externalizing problems (*T*-score subscale)	ASR	48.53 (9.38)	1 (1.2)
	Psychiatric diagnosis (%)	Self-report	12 (14.8)	–
	Disability (%)	Self-report	12 (14.8)	–
Caregiving situation characteristics				
	Total number of children	Self-report	2.35 (1.01)	2 (2.5)
	Two-parent household (% partner present)	Self-report	69 (93.2)	–
	Employment caregiver (%)	Self-report	61 (75.3)	–
	Weekly working hours caregiver (total)	Self-report	17.59 (14.03)	1 (1.2)
	Employment partner (% of total partners)	Proxy report	68 (95.8)	10 (8.1)
	Weekly working hours caregiver’s partner (total)	Proxy report	37.07 (12.77)	10 (8.1)
	Family functioning (total score)	FAD	22.08 (4.58)	5 (6.2)

*IQ* intelligence Quotient, *SRS* Social Responsiveness Scale, *ADOS-2 CSS* Autism Diagnostic Observation Schedule-2 Calibrated Severity Scale, *CBCL* Child Behavior Checklist, *INT* Internalizing, *EXT* Externalizing, *RBS-R* Repetitive Behavior Scale-Revised, *CERQ* Cognitive Emotion Regulation Questionnaire, *PGS* Personal Growth Scale, *PR* Partner Relationship, *SC* Social Contacts, *SRS-A* Social Responsiveness Scale-Adults, *ASR* Adult Self-Report, *FAD* Family Assessment Device, *OBVL* Opvoedingsbelasting Vragenlijst [Parenting Stress Questionnaire]

### Associations with Caregivers’ Health-Related QoL

In univariate single variable regression analysis (Table S1), higher health-related QoL was significantly associated with child variables such as an younger age (*p* = 0.028), a lower autism severity (*p* = 0.024), and less repetitive behavior problems/interests (*p* = 0.021). Also, higher health-related QoL was significantly associated with caregiver variables such as male sex (*p* = 0.016), a higher educational level (*p* = 0.033), and a more adaptive coping style (*p* = 0.028), less social impairment (*p* = 0.005), less internalizing problems (*p* < 0.001), less externalizing problems (*p* = 0.002), not having a psychiatric diagnosis (*p* = 0.001), and less parenting stress (*p* = 0.002). Among the caregiving situation variables, more weekly working hours of the caregiver (*p* = 0.003), and employment of the caregiver’s partner (*p* = 0.007) were significantly associated with a higher health-related QoL.

Next, we included the variables with *p* < 0.05 in the stepwise regression model (Table 2) of the caregiver’s health-related QoL (EQ-5D). The resulting model with the caregiver variables internalizing problems (*p* < 0.001) and adaptive coping (*p* = 0.009) explained 38% of the variance of the health-related QoL. To test the robustness of the results, we also performed the ‘forced entry’ multivariable regression procedure in this step. Using this procedure, we found similar results. However, the adaptive coping was no longer significantly associated. There were no significant differences between the caregivers included in the multivariable regression analysis (*n* = 71) and the caregivers with missing values (*n* = 10), except for a higher percentage of children with Dutch ethnicity of the included caregivers (*p* = 0.023).

**Table 2:** Multivariable regression analysis with the health-related QoL (EQ-5D) as dependent variable among caregivers of children with an ADOS-2 ASD classification (significant factors from univariate single variable regression analyses with p ≤ 0.05)

Variable	*B*	95% CI for B		*SE B*	*ẞ*	*p*	*R*^*2*^	*Adj. R*^*2*^
		LB	UB					
Model (*n* = 71) Constant	1.078	0.892	1.263	0.093		< 0.001	0.397	0.379**
*Child general variables*								
Age child								
*Child problem and psychopathology variables*								
Autistic traits								
Repetitive behavior								
*Caregiver general variables*								
Sex caregiver								
Education level								
Adaptive coping- putting into perspective	0.011	0.003	0.020	0.004	0.258**	0.009		
Parenting stress								
*Caregiver problem and psychopathology variables*								
Social impairment								
Internalizing problems	− 0.007	− 0.010	-0.005	0.001	− 0.541**	< 0.001		
Externalizing problems								
Psychiatric diagnosis								
*Caregiving situation variables*								
Weekly hours working caregiver								
Employment caregiver’s partner								

*CI* confidence interval, *LB* lower bound, *UB* upper bound

*p < 0.05

**p < 0.01

### Associations with Caregivers’ Care-Related QoL

In single-variable regression analysis (Table S2), caregiver’s variables, such as more adaptive coping (*p* = 0.024), less maladaptive coping (*p* = 0.001), more partner support (*p* = 0.005), more social support (*p* = 0.008), less internalizing problems (*p* < 0.001), less externalizing problems (*p* = 0.021), not having a psychiatric diagnosis (*p* = 0.005), less parenting stress (*p* < 0.001), and a caregiving situation variable, viz. better family functioning (*p* < 0.001), were associated with a higher care-related QoL. Among the child variables, we also found higher care-related QoL to be significantly associated with less social impairment of the child (*p* = 0.031).

Next, with a stepwise regression analysis, entering the variables which were significantly associated in the univariate analysis with the care-related QoL of the caregiver (*p* < 0.05) (Table 3). The model that included adaptive coping style (*p* < 0.001) and parenting stress (*p* < 0.001) was found to explain 60% of the variance of the care-related QoL. Caregivers included in the multivariable regression analysis (*n* = 66) showed a higher percentage of children with Dutch ethnicity (*p* = 0.019) and higher parenting stress (*p* = 0.010) compared to those not included due to missing values (*n* = 15). With the ‘forced entry’ multivariable regression procedure, to test the robustness of the results, we did not find different results.

**Table 3:** Multivariable regression analysis with the Care-related QoL (CarerQol) as dependent variable among caregivers of children with an ADOS-2 ASD classification (significant factors from univariate single variable regression analyses with *p* ≤ 0.05)

Variable	*B*	95% CI for B		*SE B*	*β*	*p*	*R*^*2*^	*Adj. R*^*2*^
		LB	UB					
Model (*n* = 66) Constant *Child variables* Social impairment	109.467	94.079	124.856	7.701		< 0.001	0.612	0.600**
*Caregiver general variables*								
Adaptive coping	1.559	0.833	2.285	0.363	0.337**	< 0.001		
Maladaptive coping								
Partner-relationship								
Social support								
Parenting stress	− 0.827	− 1.015	− 0.640	0.094	− 0.693**	< 0.001		
*Caregiver problem and psychopathology variables*								
Internalizing problems								
Externalizing problems								
Psychiatric diagnosis								
Caregiving situation variables								
Family functioning								

*CI* confidence interval, *LB* lower bound, *UB* upper bound

*p < 0.05

**p < 0.01

## Discussion

The present study is the first to simultaneously investigate associations of several child, caregiver, and caregiving situation variables with both health-related and care-related QoL in caregivers of clinically referred children with an ASD classification. Because previous research mostly focused on such associations with a restricted set of variables, we attempted to explore associations of a rich set of relevant independent variables with the caregiver’s QoL. In our study sample, child variables did not seem to be associated with the caregiver’s QoL, if caregiver and caregiving situation variables were taken into account. With including two unique perspectives on caregiver’s QoL (i.e. health- versus care-related QoL), we hypothesized that these two outcome variables might each uniquely be associated with different variables. Our study results support this hypothesis partially, with higher *health-related* QoL associated with less caregiver’s internalizing problems and more caregiver’s adaptive coping, and higher *care-related* QoL associated with less caregiver’s parenting stress and more caregiver’s adaptive coping. These associated variables together explained a substantial part of the variance in the health- and care-related QoL, 38% and 60%, respectively. To provide information relevant to improving the QoL of caregivers of children with ASD, we will address some study results in more detail.

Child variables were not significantly associated with the caregiver’s health-and care-related QoL in our study when caregiver and caregiving situation variables were taken into account. Vasilopoulou and Nisbet (2016) already concluded that over half of the reviewed studies did not detect an association between autism severity and caregiver’s QoL. In contrast to our results, they found more studies with an inverse association between externalizing (behavior) problems of the child and the caregiver’s QoL. Variations in the used QoL instruments, the child factors, and study samples, but also the measurement of other variables, could account for the different results. In comparison, Jain et al. (2018) also did not find a relation between care-related QoL (on the CarerQol) and seizure severity in caregivers of children and adolescents with drug-resistant epilepsy, including caregiver and caregiving situation variables. However, in a group of young children with cystic fibrosis, Fitzgerald et al. (2018) reported a higher care-related QoL (on the CarerQol) in caregivers to be related to less disease severity, next to younger child age, and being a father. In this study, fewer caregiver and caregiving situation variables were included. Our study suggests that strategies to improve the caregiver’s QoL should predominantly be targeted at the associated caregiver’s and caregiving variables instead of child variables.

The finding of an inverse, significant association between the caregiver’s health-related QoL and self-reported internalizing problems, is in line with previous results in caregivers of children with ASD (Allik et al., 2006; Khanna et al., 2013; Kuhlthau et al., 2014). In line with other studies, ninety percent of the caregivers in our study were female. Importantly, Khanna et al. (2013) found that female caregivers of children with ASD reported lower health-related QoL than females in the general population. Hastings et al. (2005) reported more depression in female caregivers than in male caregivers of children with ASD. Thus, all these findings suggest that caregivers of children with ASD seem to be at risk for internalizing problems (i.e., anxiety, depression, withdrawal, and somatic complaints in our study) and lower health-related QoL. This is important to consider when starting parent guidance as part of the treatment of children with ASD (Volkmar et al., 2014). After all, there is a continuous reciprocal interaction process between children and their caregiver, in which the caregiver’s well-being is crucial for the successful application of interventions (Osborne et al., 2008; Rodriguez et al., 2019). A potential causal relationship might be bi-directional: caring for a child with ASD is reported to be demanding and stressful, which elevates the risk of developing internalizing problems and, as a consequence, lowering the QoL in caregivers (Kuhlthau et al., 2014). Vice versa, a lower health-related QoL can cause more internalizing problems and, as a consequence, or because of the reciprocity between a child and the caregiver, more perceived stress in raising the child with ASD. Also, a genetic predisposition of internalizing problems in caregivers of children with ASD was suggested before (Duvekot et al., 2016; Mugno et al., 2007). The causality and potential conceptual overlap between the health-related QoL and internalizing problems of caregivers need to be investigated further.

In the present study, only adaptive coping was significantly associated with both more health- and care-related QoL. Some previous studies focused on the association of coping with the caregiver’s QoL, mostly maladaptive coping (Khanna et al., 2011; Lee et al., 2009), and were inconclusive because of differences in study samples and coping instruments. By using the CERQ for the caregiver’s coping, we focused on cognitive emotion regulation (Garnefski & Kraaij, 2007) instead of a broader coping construct with, for example, behavioral coping included. Interestingly, our finding points to the enhancement of adaptive coping skills for caregivers as a possible strategy to improve the QoL. However, the found association might also be bi-directional. Furthermore, we need to be cautious when interpreting this result because in testing the robustness of the results, the association between adaptive coping and the health-related QoL was no longer statistically significant when applying a different procedure of multivariable regression analysis (‘forced entry’). Further studies should be directed in replicating our study results, as well as exploring causality and potential conceptual overlap between coping and QoL.

The partner’s employment seemed to be the only caregiving situation variable associated with the health-related QoL, but was no longer associated at a significance level of 0.01%. Nevertheless, this is an interesting finding suggesting that the partner’s employment status could affect the caregiver’s health-related QoL, possibly because of less financial problems or burden. Kuhlthau et al. (2014) demonstrated previously the impact of the financial burden of families related to caring for children with ASD. Bittman et al. (2007) found in a sample of caregivers for heterogeneous care recipients, that male caregivers were less likely to give up fulltime employment than female caregivers with informal caring. Since our sample contained predominantly female caregivers, possibly the partner's employment might be a more relevant factor. On the other hand, higher QoL of the primary caregiver could also be associated with the probability of partners being employed because of higher socio-economic status, educational level, and fewer family problems at home. Unfortunately, income data were lacking in this study. Other employment variables, such as the caregiver’s employment status and weekly working hours, were not associated with the care-related QoL in our study. Further studies could explore if and how these caregiving situation variables are associated with caregiver’s health-related QoL.

As expected, we found partly different variables associated with the health- and care-related QoL, confirming two distinct perspectives on caregiver’s QoL. Only caregiver variables, and neither child nor caregiving situation variables, were associated with both *health*- and *care-related* QoL in our study. As hypothesized in the study sample, care-related QoL seems to be especially related to the caring tasks of the caregiver, whereas the health-related QoL is related to the own (mental) health. This also highlights that the choice of QoL instrument instruments should be made carefully, in relation to the perspective, expected outcome, and the specific research or clinical questions at hand.

### Strengths and Limitations

The fact that the collected data were part of a unique rich data-set with many child, caregiver, as well as caregiving situation variables is a strength of the study. We were able to simultaneously explore the associations of all variables with the health-related QoL, and for the first time, also with the care-related QoL. Another strength is the inclusion of a well-defined group of children with the use of the ADOS-2, a gold standard instrument in clinical and research practice. Finally, the most strongly associated variables explained much of the variance within the caregiver’s QoL.

However, the study results should be interpreted in the context of the following limitations. The study sample size was limited, also in relation to the statistical analyses performed. In light of the study's exploratory nature and the clinical importance of investigating the associations of all these child, caregiver, and caregiving situation variables on the caregiver’s QoL, we did not limit the number of variables. Still, we used a significance level of 0.01% to reduce multiple testing effect. With this adaptation, we found no differences concerning the associations with the care-related QoL, but partner’s employment was no longer significantly associated with health-related QoL To overcome possible multicollinearity between variables and confounding, we tested and met the underlying assumptions of the regression models. Moreover, we used a stepwise procedure of the multivariable regression analyses to minimize this risk. With a different regression analysis procedure (‘forced entry’), to test the robustness of the results, we found no differences concerning the associations for care-related QoL, but also adaptive coping was no longer significantly associated with health-related QoL. These association should be further explored in future studies.

Concerning the generalizability of the results, we have to consider how representative the study sample is. The included caregivers were caring for a clinically referred group of children, which could imply a possible bias of more affected children and caregivers experiencing more problems compared to other study samples (Hoefman et al., 2014; Khanna et al., 2013). Although our caregivers seemed to be rather well-off with high quality of life (mean EQ-5D utility index 0.84; mean CarerQol sum tariff 77.33), mostly employed (75%), highly educated (26%), and almost all were living with their child in a two-parent household (93%), these characteristics are in line with the other caregiver study samples. Compared to the aforementioned studies, the children in our study appeared to be slightly less intellectually impaired and had fewer autism traits, as reported on the SRS (Table 1). The young age range (2–10 years) may have constrained the variability of impact on the caregiver’s QoL. Presumably, the effect of child factors, such as autism traits and behavioral problems, may increase with older age because of complexity that increases during adolescence. Tung et al. (2014) found a slight age effect on the caregiver’s QoL, but this was not confirmed in other studies that (also) included adolescents (Kuhlthau et al., 2014; Lee et al., 2009).

Another limitation is the fact that the caregivers reported almost all child, caregiver, caregiving variables, and QoL aspects. Some associations between the variables and caregiver’s QoL may be affected by originating from the same informant (Duvekot et al., 2016), although we did not find this association between the caregiver’s reports of the child variables and caregiver’s QoL. We preferred the caregiver’s proxy reports of the child variables instead of self-reports, because of the age range in our study (2–10 years), possible limitations in reporting because of ASD (i.e., limited self-reflection capacities), and the use of one version of instruments in the whole sample. To partly overcome this, we used the clinical assessment of trained and certified psychologists in classifying the ASD in the children (using the ADOS-2). Including information from other sources, such as self-report of the secondary caregivers (i.e., informal caregivers, second-most involved in the caring for the child, mostly the fathers), would be interesting in future studies (Allik et al., 2006; Mugno et al., 2007).

### Clinical Implications

The exploration of which variables are associated with the caregiver’s health- and care-related QoL, indicates the importance of investing in improving the caregiver’s mental health, especially concerning internalizing problems, training caregivers in developing adaptive coping skills, and diminishing parenting stress, in the treatment and guidance of children with ASD. The study results provide potential ingredients for several strategies to improve the caregiver’s QoL in the care for children with ASD. With the different perspectives, it is important to realize which QoL perspective to use in targeting improvement of the caregiver’s QoL. In implementing treatment plans for the children, it may be useful to assess the caregiver’s characteristics such as coping styles, parenting stress, and mental health problems (especially anxiety, depressive symptoms, somatic complaints). Most treatment plans are often entirely focused on training the child’s (social) skills and diminishing the child’s behavior problems; these strategies are not targeted at improving the caregiver’s QoL. However—and in line with the results of this study—Catalano et al. (2018) recommended, in support guidelines for caregivers, to address the state of the mental health and psychological well-being of the caregivers, for example, in special care for adaptive coping skills, such as problem-solving and self-perspective taking. They stated that treatment plans should contain caregiver education, training, and if necessary, therapy sections to be more productive. Implementation of such practical guidelines may lead to improved caregiver’s QoL, which in turn is expected to—over time—have an advantage for the child and family well-being, and family resilience (Fong et al., 2021; Smith et al., 2008). Further research in more representative, more diverse, and more extensive study samples with information of both primary and secondary caregivers, is necessary to validate and replicate the current study results. Also, including a child and adolescent self-report, if possible, would be interesting. Clinical diagnostic assessment of the caregivers concerning internalizing problems, for example, anxiety and mood disorders, would also add to the knowledge. Finally, the intervention effect of education or training of caregivers with parenting stress or mental health problems compared to caregivers of non-referred children without parenting stress or mental health problems would be interesting.

### Conclusion

In this study, we found health-related QoL of caregivers to be significantly associated with self-reported internalizing problems and adaptive coping, and care-related QoL with parenting stress and adaptive coping. Health- and care-related QoL each provided a unique perspective on the caregiver’s QoL, with adaptive coping being a common factor. When simultaneously exploring child, caregiver, and caregiving situation variables of QoL, caregiver variables,—but not, as often assumed, child and caregiving situation factors—were associated. Despite the exploratory character and limitations of this study, these findings indicate the importance of investing in the caregiver’s mental health and adaptive coping styles, as well as diminishing parenting stress in the care for children with ASD.

## Supplementary Information

Below is the link to the electronic supplementary material.

Supplementary file1 (DOCX 61 kb)
