# Supplementary material for: Caring for Children with an Autism Spectrum Disorder: Factors Associating with Health- and Care-Related Quality of Life of the Caregivers
Source: J Autism Dev Disord. 2021 Nov 1;52(11):4665–78. doi: 10.1007/s10803-021-05336-7 (PMC9556348; doi:10.1007/s10803-021-05336-7)
Supplement: Supplementary file 1 — Supplementary file1 (DOCX 61 kb) [file 10803_2021_5336_MOESM1_ESM.docx]

**Caring for children with an autism spectrum disorder: factors associating with health- and care-related quality of life of the caregivers - Supplementary material**

**Table S1** Univariate single variable regression analyses of all independent child, caregiver, and caregiving situation variables with health-related QoL (EQ-5D)- as dependent variable among caregivers of children with an ADOS-2 ASD classification

| Variables (instruments) | EQ-5D |  |  |  |  |  |
| --- | --- | --- | --- | --- | --- | --- |
|  | *B* | *SE* | *t* | *p* | 95% CI | *n* |
| *Child general variables* |  |  |  |  |  |  |
| Sex child | 0.052 | 0.051 | 1.020 | 0.311 | [-0.05, 0.154] | 81 |
| Age child | -0.019* | 0.008 | -2.240 | 0.028 | [-0.036, -0.002] | 81 |
| Ranking child | 0.033 | 0.020 | 1.685 | 0.096 | [-0.006, 0.073] | 80 |
| Full-scale IQ | 0.000 | 0.001 | 0.137 | 0.891 | [-0.002,0.002] | 74 |
|  |  |  |  |  |  |  |
| *Child problem and psychopathology variables* |  |  |  |  |  |  |
| Social impairments (SRS) | -0.002 | 0.002 | -1.043 | 0.300 | [-0.005,0.002] | 81 |
| Autistic traits (ADOS-2) | -0.024* | 0.010 | -2.302 | 0.024 | [-0.045,-0.003] | 81 |
| Internalizing problems (CBCL) | -0.002 | 0.002 | -0.888 | 0.378 | [-0.007, 0.003] | 76 |
| Externalizing problems (CBCL) | -0.001 | 0.002 | -0.649 | 0.518 | [-0.005, 0.003] | 76 |
| Repetitive behavior (RBS-R) | -0/003* | 0.001 | -2.359 | 0.021 | [-0.006, 0.000] | 81 |
| Disability | -0.056 | 0.046 | -1.211 | 0.229 | [-0.148, 0.036] | 81 |
|  |  |  |  |  |  |  |
| *Caregiver general variables* |  |  |  |  |  |  |
| Sex caregiver | -0.154* | 0.063 | -2.459 | 0.016 | [-0.279, -0.029] | 81 |
| Age caregiver | 0.002 | 0.004 | 0.498 | 0.620 | [-0.006, 0.009] | 81 |
| Education level | 0.056* | 0.026 | 2.167 | 0.033 | [0.005, 0.108] | 80 |
| Adaptive coping (CERQ) | 0.011* | 0.005 | 2.245 | 0.028 | [0.001, 0.020] | 81 |
| Maladaptive coping (CERQ) | -0.009 | 0.006 | -1.599 | 0.114 | [-0.021, 0.002] | 81 |
| Personal growth (PGS) | 0.003 | 0.004 | 0.078 | 0.488 | [-0.005, 0.011] | 81 |
| Partner-relationship support (PR) | 0.009 | 0.006 | 1.594 | 0.115 | [-0.002, 0.021] | 74 |
| Social support (SC) | 0.011 | 0.006 | 1.791 | 0.077 | [-0.001, 0.024] | 81 |
| Parenting stress (OBVL) | -0.004** | 0.001 | -3.200 | 0.002 | [-0.007, -0.002] | 81 |
|  |  |  |  |  |  |  |
| *Caregiver problem and psychopathology variables* |  |  |  |  |  |  |
| Social impairments (SRS-A) | -0.006** | 0.002 | -2.901 | 0.005 | [-0.011, -0.002] | 81 |
| Internalizing problems (ASR) | -0.008** | 0.001 | -6.733 | <0.001 | [-0.010, -0.006] | 80 |
| Externalizing problems (ASR) | -0.006** | 0.002 | -3.137 | 0.002 | [-0.010, -0.002] | 80 |
| Psychiatric diagnosis | -0.182** | 0.051 | -3.579 | 0.001 | [-0.283, -0.081] | 81 |
| Disability | -0.087 | 0.054 | -1.612 | 0.111 | [-0.194, 0.020] | 81 |
|  |  |  |  |  |  |  |
| *Caregiving situation variables* |  |  |  |  |  |  |
| Total number of children | 0.029 | 0.019 | 1.469 | 0.146 | [-0.010, 0.067] | 79 |
| Partner present | 0.041 | 0.082 | 0.500 | 0.619 | [-0.122, 0.204] | 74 |
| Employment caregiver | 0.059 | 0.045 | 1.331 | 0.187 | [-0.029, 0.148] | 81 |
| Weekly working hours caregiver | 0.004** | 0.001 | 3.061 | 0.003 | [0.001, 0.007] | 80 |
| Employment caregiver’s partner | 0.256** | 0.093, | 2.763 | 0.007 | [0.071, 0.441] | 71 |
| Weekly working hours caregiver’s partner | 0.001 | 0.002 | 0.401 | 0.690 | [-0.002, 0.004] | 71 |
| Family functioning (FAD) | -0.008 | 0.004 | -1.911 | 0.060 | [-0.017, 0.000] | 76 |
|  |  |  |  |  |  |  |

*p < 0.05; **p < 0.01

*CI* confidence interval, *IQ* Intelligence Quotient, *SRS* Social Responsiveness Scale, *ADOS-2 CSS* Autism Diagnostic Observation Schedule-2, *CBCL* Child Behavior Checklist, *RBS-R* Repetitive Behavior Scale-Revised, *CERQ* Cognitive Emotion Regulation Questionnaire, *PGS* Personal Growth Scale, *PR* Partner Relationship, *SC* Social contacts, *OBVL* Opvoedingsbelasting vragenlijst [Parenting Stress Questionnaire], *SRS-A* Social Responsiveness Scale-Adults, *ASR* Adult Self-Report, *FAD* Family Assessment Device.

**Table S2** Univariate single variable regression analyses of all independent child, caregiver, and caregiving situation variables with care-related QoL (CarerQol) as dependent variable among caregivers of children with an ADOS-2 ASD classification

| Variables (instruments) | CarerQol |  |  |  |  |  |
| --- | --- | --- | --- | --- | --- | --- |
|  | *B* | *SE* | *t* | *p* | 95% CI | *n* |
| *Child general variables* |  |  |  |  |  |  |
| Sex child | -1.202 | 4.895 | -0.245 | 0.807 | [-10.955, 8.552] | 76 |
| Age child | -0.808 | 0.846 | -0.955 | 0.343 | [-2.494, 0.878] | 76 |
| Ranking child | 0.393 | 1.932 | 0.204 | 0.839 | [-3.458, 4.244] | 75 |
| Full-scale IQ | 0.066 | 0.106 | 0.624 | 0.535 | [ -0.146, 0.279] | 70 |
|  |  |  |  |  |  |  |
| *Child problem and psychopathology variables* |  |  |  |  |  |  |
| Social impairments (SRS) | -0.338* | 0.154 | -2.197 | 0.031 | [-0.645, -0.031] | 76 |
| Autistic traits (ADOS-2) | -0.305 | 1.062 | -0.287 | 0.775 | [-2.422, 1.812] | 76 |
| Internalizing problems (CBCL) | -0.220 | 0.220 | -0.998 | 0.322 | [-0.659, 0.220] | 72 |
| Externalizing problems (CBCL) | -0.108 | 0.189 | -0.569 | 0.571 | [-0485, 0,269] | 72 |
| Repetitive behavior (RBS-R) | -0.102 | 0.131 | -0.779 | 0.439 | [-0.362, 0,159] | 76 |
| Disability | 3.454 | 4.538 | 0.761 | 0.449 | [-5.588, 12,496] | 76 |
|  |  |  |  |  |  |  |
| *Caregiver general variables* |  |  |  |  |  |  |
| Sex caregiver | -1.849 | 7.073 | -0.263 | 0.793 | [-15.870, 12.172] | 76 |
| Age caregiver | 0.519 | 0.360 | 1.443 | 0.153 | [-0.197, 1.235] | 76 |
| Education level | 3.383 | 2.589 | 1.307 | 0.195 | [-1.775, 8.542] | 76 |
| Adaptive coping (CERQ) | 1.081* | 0.469 | 2.303 | 0.024 | [0.146, 2.016] | 76 |
| Maladaptive coping (CERQ) | -1.828** | 0.533 | -3.430 | 0.001 | [-2.890,-0.766] | 76 |
| Personal growth (PGS) | 0.469 | 0.393 | 1.192 | 0.237 | [-0.315, 1.253] | 76 |
| Partner-relationship support (PR) | 1.587** | 0.545 | 2.912 | 0.005 | [0.500, 2.674] | 70 |
| Social support (SC) | 1.616** | 0.595 | 2.715 | 0.008 | [0.430. 2.802] | 76 |
| Parenting stress (OBVL) | -0.791** | 0.098 | -8.101 | <0.001 | [-0.985, -0.596] | 76 |
|  |  |  |  |  |  |  |
| *Caregiver problem and psychopathology variables* |  |  |  |  |  |  |
| Social impairments (SRS-A) | -0.370 | 0.219 | -1.690 | 0.095 | [-0.807, 0.066] | 75 |
| Internalizing problems (ASR) | -0.717** | 0.127 | -5.662 | <0.001 | [-0.969, -0.465] | 75 |
| Externalizing problems (ASR) | -0.463* | 0.196 | -2.358 | 0.021 | [-0.855, -0.072] | 76 |
| Psychiatric diagnosis | -14.345** | 4.932 | -2.909 | 0.005 | [-24.172,-4.518] | 76 |
| Disability | -0.372 | 5.206 | -0.072 | 0.943 | [-10.746, 10.001] | 76 |
|  |  |  |  |  |  |  |
| *Caregiving situation variables* |  |  |  |  |  |  |
| Total number of children | 2.337 | 1.863 | 1.255 | 0.214 | [-1.376, 6.051] | 74 |
| Partner present | -10.109 | 8.643 | -1.170 | 0.246 | [-27.355, 7.137] | 70 |
| Employment caregiver | 4.703 | 4.276 | 1.100 | 0.275 | [-3.818 13.224] | 76 |
| Weekly working hours caregiver | 0.131 | 0.146 | 0.895 | 0.374 | [-0.161, 0.422] | 75 |
| Employment caregiver’s partner | -6.757 | 9.898 | -0.683 | 0.497 | [-26.525, 13.011] | 67 |
| Weekly working hours caregiver’s partner | -0.035 | 0.165 | -0.209 | 0.835 | [-0.364, 0.295] | 63 |
| Family functioning (FAD) | -1.448** | 0.389 | -3.723 | <0.001 | [-2.224, -0.672] | 72 |
|  |  |  |  |  |  |  |

*p < 0.05; **p < 0.01

*CI* confidence interval, *IQ* Intelligence Quotient, *SRS* Social Responsiveness Scale, *ADOS-2 CSS* Autism Diagnostic Observation Schedule-2, *CBCL* Child Behavior Checklist, *RBS-R* Repetitive Behavior Scale-Revised, *CERQ* Cognitive Emotion Regulation Questionnaire, *PGS* Personal Growth Scale, *PR* Partner Relationship, *SC* Social contacts, *OBVL* Opvoedingsbelasting vragenlijst [Parenting Stress Questionnaire], *SRS-A* Social Responsiveness Scale-Adults, *ASR* Adult Self-Report, *FAD* Family Assessment Device.
